# Clinical Features of Bacterial Vaginosis in a Murine Model of Vaginal Infection with *Gardnerella vaginalis*

**DOI:** 10.1371/journal.pone.0059539

**Published:** 2013-03-19

**Authors:** Nicole M. Gilbert, Warren G. Lewis, Amanda L. Lewis

**Affiliations:** 1 Department of Molecular Microbiology, Washington University School of Medicine, St. Louis, Missouri, United States of America; 2 Department of Medicine, Washington University School of Medicine, St. Louis, Missouri, United States of America; 3 Department of Obstetrics and Gynecology, Center for Women’s Infectious Disease Research, Washington University School of Medicine, St. Louis, Missouri, United States of America; Columbia University, United States of America

## Abstract

Bacterial vaginosis (BV) is a dysbiosis of the vaginal flora characterized by a shift from a *Lactobacillus*-dominant environment to a polymicrobial mixture including *Actinobacteria* and Gram-negative bacilli. BV is a common vaginal condition in women and is associated with increased risk of sexually transmitted infection and adverse pregnancy outcomes such as preterm birth. *Gardnerella vaginalis* is one of the most frequently isolated bacterial species in BV. However, there has been much debate in the literature concerning the contribution of *G. vaginalis* to the etiology of BV, since it is also present in a significant proportion of healthy women. Here we present a new murine vaginal infection model with a clinical isolate of *G. vaginalis*. Our data demonstrate that this model displays key features used clinically to diagnose BV, including the presence of sialidase activity and exfoliated epithelial cells with adherent bacteria (reminiscent of clue cells). *G. vaginalis* was capable of ascending uterine infection, which correlated with the degree of vaginal infection and level of vaginal sialidase activity. The host response to *G. vaginalis* infection was characterized by robust vaginal epithelial cell exfoliation in the absence of histological inflammation. Our analyses of clinical specimens from women with BV revealed a measureable epithelial exfoliation response compared to women with normal flora, a phenotype that, to our knowledge, is measured here for the first time. The results of this study demonstrate that *G. vaginalis* is sufficient to cause BV phenotypes and suggest that this organism may contribute to BV etiology and associated complications. This is the first time vaginal infection by a BV associated bacterium in an animal has been shown to parallel the human disease with regard to clinical diagnostic features. Future studies with this model should facilitate investigation of important questions regarding BV etiology, pathogenesis and associated complications.

## Introduction

One in three women in the U.S. have bacterial vaginosis (BV) [1], a microbial imbalance of the vaginal flora characterized by the absence of normally dominant lactobacilli and overgrowth of complex communities dominated by Gram-negative bacteria and *Actinobacteria* [2], [3]. BV can be asymptomatic, maybe even part of a spectrum of ‘normal’ from the patient perspective, but often displays characteristic clinical features, including “thinning” of vaginal fluid secretions, increased pH (>4.5), a fishy odor upon potassium hydroxide treatment, and the presence of clue cells (epithelial cells studded with bacteria) in wet mounts. An additional defining feature of BV is the presence of vaginal sialidase [4]–[9], an enzyme that cleaves terminal sialic acid residues from complex glycans, which are abundant on host cell surfaces and secreted mucus proteins [10]–[12]. Women with BV are at increased risk of pelvic inflammatory disease, infections following surgery or other routine gynecologic procedures, sexually transmitted infections including HIV, and serious pregnancy complications such as intrauterine infection and preterm birth [13]–[22].

Unlike most common infectious diseases, BV appears to be polymicrobial in nature. Recent genomic studies have illustrated the complexity and heterogeneity of BV, which can vary in bacterial composition from day to day and from one individual to another [2], [3], [23]–[27]. Although more than a dozen bacterial species have been associated with BV, the potential causal contributions of each to the biochemical, cellular, and clinical features of BV remain elusive. *Gardnerella vaginalis* was the first bacterium implicated in the pathogenesis of BV and continues to be associated with the disease [28]. However, there has been much debate in the literature concerning the contribution of *G. vaginalis* to the development and pathogenesis of BV. *G. vaginalis* can be isolated/detected from asymptomatic women that do not meet the criteria for BV diagnosis at the time of detection [2], [27], [29]–[31], causing some to question its potential role in BV. However, consistent with the notion of *G. vaginalis* as a potential pathogen, strains identified as *G. vaginalis* have been isolated from invasive perinatal infections [32]–[34]. Moreover, several investigations have described the pathogenic potential of some *G. vaginalis* isolates in cell adhesion and entry, cytolytic toxin production, biofilm formation, and other phenotypes that may reflect virulence [26], [35]–[37].

One important diagnostic feature of BV is the presence of clue cells, which are thought to be exfoliated epithelial cells coated with bacteria. *G. vaginalis*, among other BV-associated bacteria, has been shown to interact with vaginal epithelial cells in culture [37], [38], and clinical studies have shown that vaginal specimens from women with BV have adhered bacteria on their surfaces [36], [39]–[42]. However, experimental investigation of the potential role of *Gardnerella vaginalis* in generating clue cells *in vivo* requires an animal model with features of human BV.

Upon infectious challenge, epithelial cells within the urinary and genital tracts can undergo a process termed exfoliation, in which superficial cells appear to be actively shed from the epithelial surface. In some cases, this is beneficial to the host, flushing potential pathogens from the mucosa [43], [44], while in other cases, potential pathogens can take advantage of their access to underlying mucosal tissue [45], [46]. Despite much speculation in the medical literature regarding the potential causes of epithelial exfoliation in BV, including enzymes such as sialidase and prolidase or the combination of amines and organic acids produced by anaerobes [37], [47]–[50], the degree of vaginal epithelial shedding among women with BV or normal flora does not appear to have been directly measured or reported in the clinical literature. Distinguishing whether epithelial exfoliation is actively induced in BV is necessary for establishing the pathophysiology of the disease and may be important for understanding why women with BV are at increased risk of secondary urogenital and intrauterine infections.

Defining a role for *G. vaginalis* (or other associated bacteria) in BV pathogenesis has been hampered by the absence of robust small animal models that displays phenotypes seen in human BV. Here we describe the development of a new murine model of *G. vaginalis* vaginal infection. This model displays several key features of BV, including presence of vaginal sialidase activity, and the presence of epithelial cells with attached bacteria (reminiscent of clue cells). Additionally, we provide the first quantitative evaluation of vaginal epithelial exfoliation in BV, demonstrating increased shedding of epithelial cells in both *G. vaginalis* infected mice and in clinical specimens from women with BV compared to mock-infected mice or women with normal flora respectively. This epithelial response is contrasted by an absence of inflammatory cell infiltrate, consistent with the lack of vaginal inflammation found in human BV throughout the literature [51]–[53]. The results from this murine model suggest that *G. vaginalis* alone is sufficient to yield BV phenotypes and provide further justification for considering *G. vaginalis* as a contributor to the causes and complications associated with BV.

## Results

### Murine Vaginal and Ascending Uterine Infection by *G. vaginalis* Occurs in the Absence of an Overt Inflammatory Infiltrate

**Figure 1:**
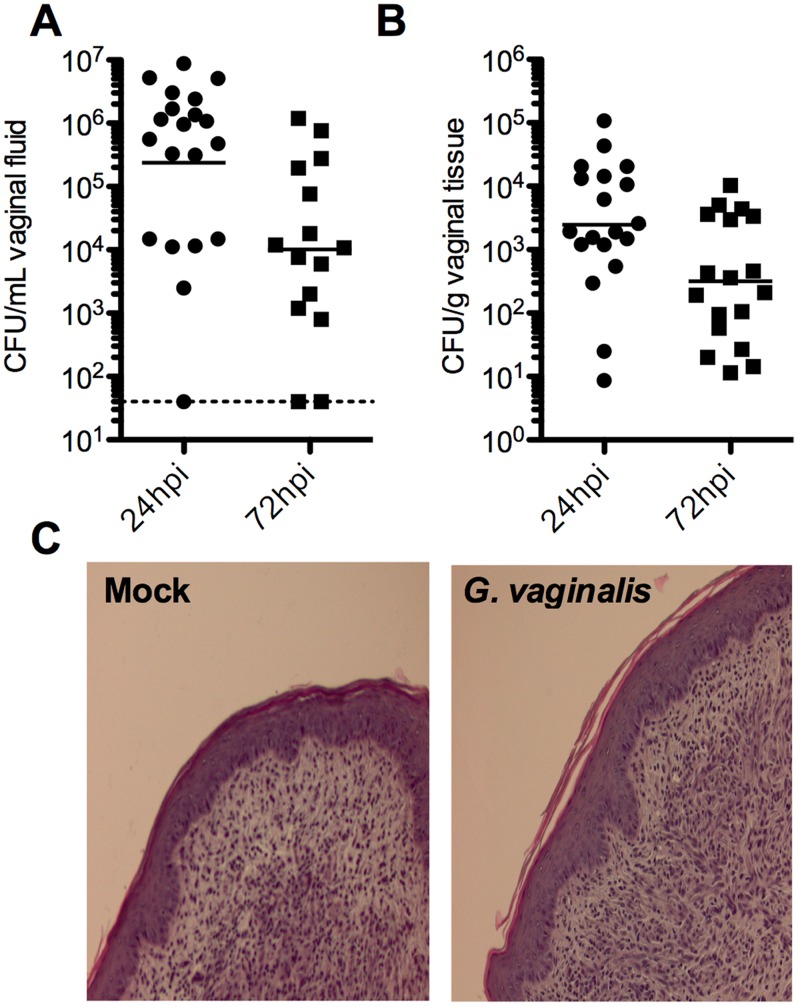
Vaginal infection by *G. vaginalis* results in minimal histological inflammation. *G. vaginalis* JCP8151B-Sm^R^4 titers were determined by enumerating colony forming units (CFU) in vaginal washes (A) and tissue homogenates (B) at 24 and 72 hpi. For samples containing no colonies, the limit of detection for each was determined and is displayed instead of a value of 0. Results are meta data from 2 independent experiments, each with 10 mice/infection group. (C) Histological inflammation was assessed by hematoxylin-eosin (H&E) staining of formalin-fixed, paraffin-embedded vaginal tissue sections.

To establish a *G. vaginalis* murine vaginal infection model, we used a sialidase-positive *G. vaginalis* strain (JCP8151B), a clinical isolate from a woman with BV. From our initial experiments we found that the majority of β-estradiol-treated C57/Bl6 mice contained vaginal flora producing large, mucoid colonies on *Gardnerella* semi-selective media that occluded the smaller *G. vaginalis* colonies. These ‘contaminating’ vaginal flora colonies were resistant to the addition of a combination of sulfamethoxazole, trimethoprim sulfate, gentamicin and perfloxacin to selection plates. Previous vaginal models of *G. vaginalis* did not acknowledge the presence of endogenous flora or describe methods by which *G. vaginalis* was distinguished and measured [54], [55]. We performed sequencing of genes encoding 16S rRNA from isolated colonies of the murine vaginal flora and identified several isolates to be species of *Enterococcus* (*faecalis* and *gallinarum*). To circumvent this issue and enable accurate determination of *G. vaginalis* titers in the murine reproductive tract, we isolated a spontaneous streptomycin resistant (Sm^R^) mutant of JCP8151B. We confirmed that the Sm^R^ isolate contained the canonical mutation in the Rpsl gene (K43N) and displayed logarithmic growth rate and sialidase activity indistinguishable from the parent strain (data not shown). Using this new JCP8151B-Sm^R^ strain, we inoculated *G. vaginalis* vaginally into C57/Bl6 mice and determined CFU levels in vaginal washes and vaginal homogenates at 24 and 72 hours post infection (hpi) (**Fig. 1A–B**). Colonies presumed to be *G. vaginalis* due to growth on streptomycin-containing plates were also confirmed by PCR using primers reported to be specific for *G. vaginalis* [56] (data not shown).

BV has been characterized clinically as a microbial condition that most often lacks obvious signs of inflammation in vaginal tissue [57]. To determine the extent of histological inflammation (edema, neutrophil infiltrate) in our murine model, we performed hematoxylin and eosin (H&E) staining on vaginal tissues collected at 24 and 72 hpi. Similar to clinical observations of BV, *G. vaginalis* infection did not result in marked tissue inflammation, edema, or polymorphonuclear (PMN) cell infiltrate (**Fig. 1C**). The mean CFU levels in both vaginal washes and homogenates decreased significantly from 24 hpi to 72 hpi (**Fig. 1A–B**), suggesting clearance of the bacteria is occurring by PMN-independent mechanisms.

**Figure 2:**
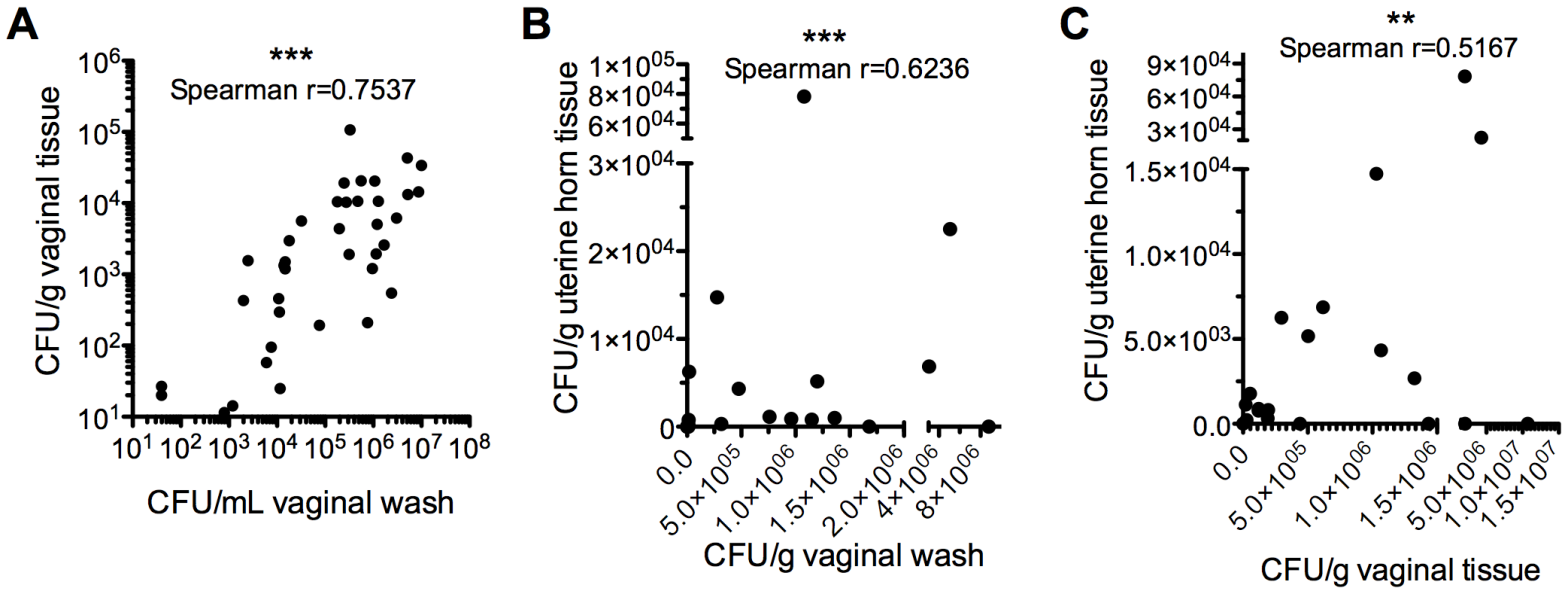
*G. vaginalis* titers in vaginal washes reflect titers in vaginal tissue and correlate directly with titers of *G. vaginalis* in uterine horns. Vaginal wash and tissue titers determined as in Fig. 1 were plotted and analyzed using GraphPad® Prism 5.0. ***P<0.001; *P<0.05. Results are meta data from 2 independent experiments, each with 10 mice/infection group.

There was a strong positive correlation between CFU determined from vaginal washes and vaginal homogenates (**Fig. 2A**). Additional experiments determined that *G. vaginalis* could persist in the murine vagina of ∼50% of mice for as long as 8 days post infection (data not shown). *G. vaginalis* was also found at low levels (mean 69.5 CFU/g) in the uterine horns of 55% and 45% of mice at 24 and 72 hpi, respectively. As might be expected, animals with higher titers of vaginal bacteria were more likely to exhibit ascending infections of the uterine horns (**Fig. 2B–C**).

### *G. vaginalis* Leads to Vaginal Sialidase Activity in vivo

**Figure 3:**
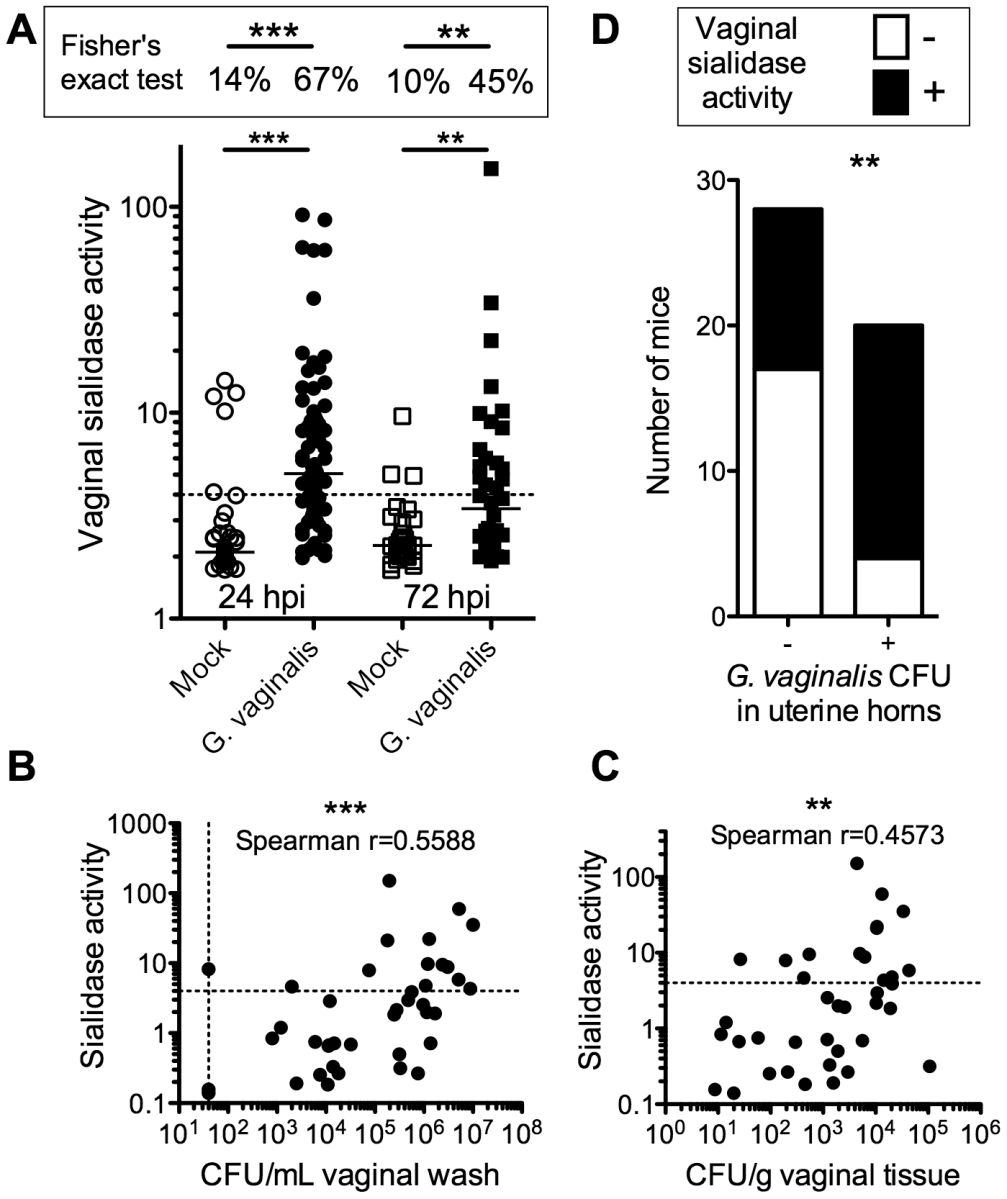
Vaginal sialidase activity correlates with *G. vaginalis* vaginal infection and is associated with ascending uterine horn infection. (A) Sialidase enzymatic activity was determined in vaginal washes from mock and *G. vaginalis* infected mice at 24 and 72 hpi and statistical significance determined by the Mann-Whitney test. The dotted line represents the cutoff value used to distinguish sialidase (+) from sialidase (−) vaginal washes for performing Fisher’s exact test, shown in the box above. Results are meta data from 4 independent experiments (24 hpi-mock n = 44, *G. vaginalis* n = 70; 72 hp-mock n = 29, *G. vaginalis* n = 40). The sialidase activity value from each *G. vaginalis*-infected mouse was plotted against *G. vaginalis* CFU in the corresponding vaginal wash (B) or tissue homogenate (C) and Spearman correlations were determined. ***P<0.0001; **P<0.005. (D) Fisher’s exact test to determine whether ascending uterine horn infection by *G. vaginalis* was contingent upon the presence of vaginal sialidase activity. Results shown represent combined data from both 24 and 72 hpi, though each time point was significant when analyzed individually.

A hallmark feature of bacterial vaginosis is the presence of high levels of sialidase activity in vaginal fluid compared to specimens from women with normal flora. Our JCP8151B-Sm^R^4 isolate produces robust sialidase activity in culture. To determine whether JCP8151B-Sm^R^4 expresses sialidase activity during vaginal infection, we performed sialidase activity assays on vaginal washes collected above. Sialidase activity was present in washes from 67% of *G. vaginalis* infected mice at 24 hpi, while the majority (86%) of mock-infected mice contained no detectable sialidase activity (**Fig. 3A**). Isolation of vaginal bacteria from the few mock-infected animals with vaginal sialidase activity demonstrated that these mice were colonized with sialidase-positive *Eubacteria consortium* or *Enterococcus spp.* (data not shown). The level of sialidase activity present in washes of infected animals correlated positively with *G. vaginalis* CFU levels in vaginal washes and homogenized vaginal tissue, strongly suggesting that the observed sialidase activity in infected animals is in fact produced by *G. vaginalis* (**Fig. 3B and C**). Together these results strongly suggest that *G. vaginalis* expresses sialidase in the murine vagina and for the first time establish a prominent biochemical feature of BV in a murine infection model.

A greater percentage of mice displaying vaginal sialidase activity also had *G. vaginalis* in their uterine horns than those mice that were sialidase negative (**Fig. 3D**). These data are consistent with the observation that higher vaginal titers increase the propensity for ascending infection.

### *G. vaginalis* Interacts with Murine Vaginal Epithelial Cells in vitro and in vivo

**Figure 4:**
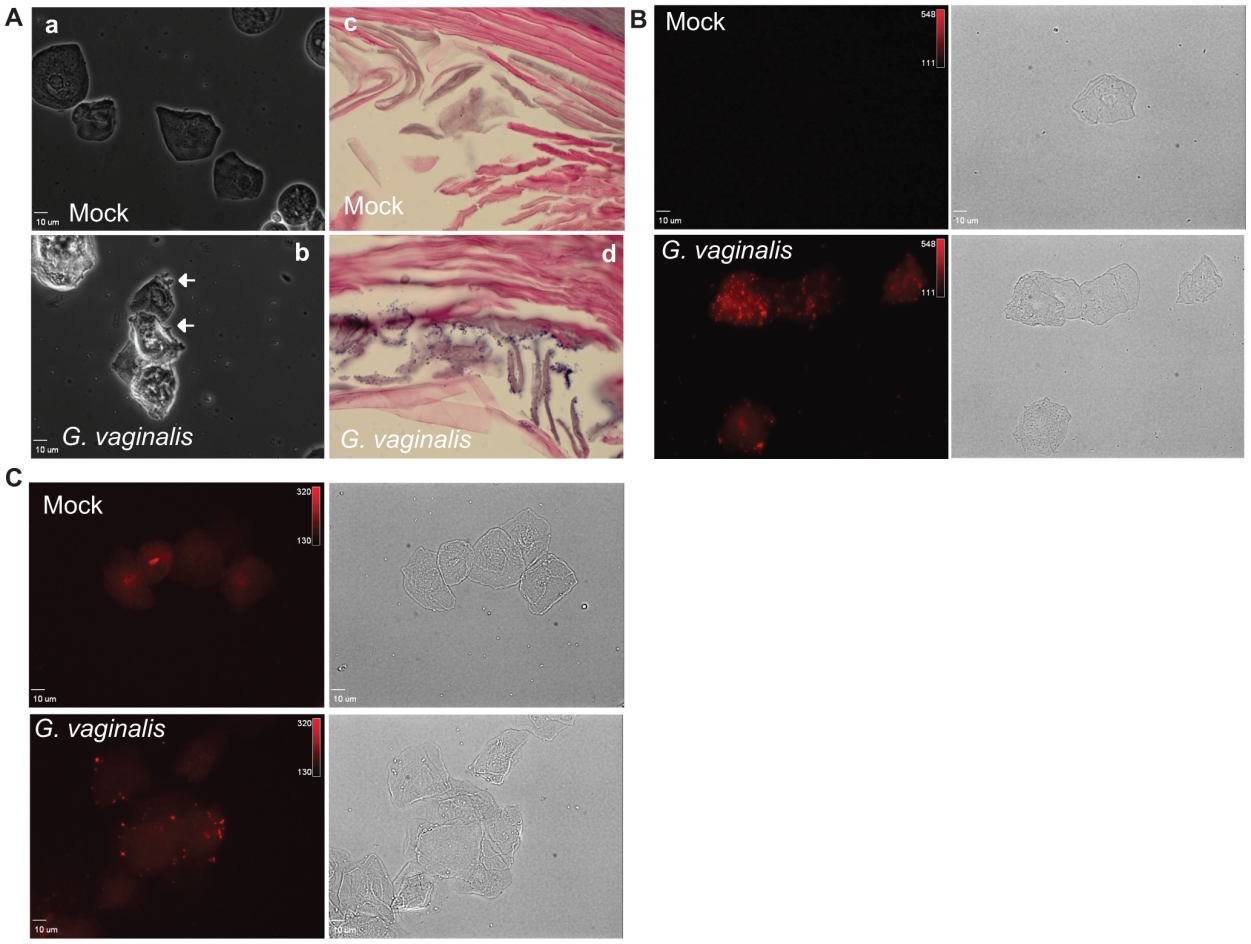
*G. vaginalis* adheres to murine vaginal epithelial cells in vitro and in vivo. (A) Phase contrast light microscopy of vaginal wash wet mounts (panels a and b) and H&E staining of vaginal tissue sections (panels c and d) from mock (a and c) and *G. vaginalis* (b and d) infected mice. Arrows in panel b and dark purple puncta in panel d illustrate potentially adherent bacteria in *G. vaginalis*-infected samples. (B) Fluorescent confocal microscopy images (with corresponding bright-field images) of vaginal epithelial cells either mock (top) or RBITC labeled *G. vaginalis* (bottom) infected in vitro for 3 h at 37°C. (C) Fluorescent confocal microscopy images of vaginal washes from mock or RBITC labeled *G. vaginalis* infected mice, collected at 4 hpi.

Another feature of BV, and a component of the Amsel criteria for BV diagnosis, is the presence of epithelial cells with adherent bacteria, termed clue cells. *G. vaginalis* has been shown to interact with cultured human vaginal epithelial cells and experiments using vaginal biopsy [42] and vaginal fluid [58] samples suggests that bacteria may form an adherent biofilm on epithelial cells in the human vagina. Vaginal washes from *G. vaginalis* infected mice often contained clumps of epithelial cells with apparent attached bacteria (**Fig. 4A**, panel b, arrowheads).

Additionally, abundant hematoxylin-rich puncta, indicative of adherent bacteria, were also apparent in some samples upon H&E staining of vaginal sections (**Fig. 4A**, panel d). These results suggested that *G. vaginalis* may interact with murine vaginal epithelial cells; however, we could not definitively distinguish *G. vaginalis* from murine vaginal flora using histology. Previously used antibodies for immunofluorescence [36] were unavailable. Therefore, to provide further confidence that *G. vaginalis* could interact with murine vaginal epithelial cells we performed infection assays with fluorescently labeled *G. vaginalis*. First we assessed adherence *ex vivo* using epithelial cells present in vaginal washes from uninfected, β-estradiol treated mice. Epithelial cells were washed extensively to remove endogenous flora, then infected with fluorescently labeled *G. vaginalis* and visualized by fluorescent confocal microscopy. *G. vaginalis* adhered to mouse vaginal epithelial cells, appearing as distinct fluorescent puncta or biofilm-like collections decorating the epithelial cell surface (**Fig. 4B**). Similar puncta were not observed on epithelial cells inoculated with a “mock” preparation (containing the fluorescent label but no bacteria), demonstrating that this pattern of fluorescence was specific to *G. vaginalis* and not an artifact of the introduction of the RBITC label. Next we determined whether we could observe such interactions during *in vivo* infection. Following vaginal inoculation with fluorescently labeled *G. vaginalis*, similar fluorescent puncta were detected on epithelial cells in vaginal washes from infected mice at 4 hpi. Together, these results demonstrate that *G. vaginalis* adheres to murine vaginal epithelial cells, similar to what has been seen for human-derived cultured epithelial cells [37], [38].

### *G. vaginalis* Infection Results in Robust Epithelial Exfoliation

**Figure 5:**
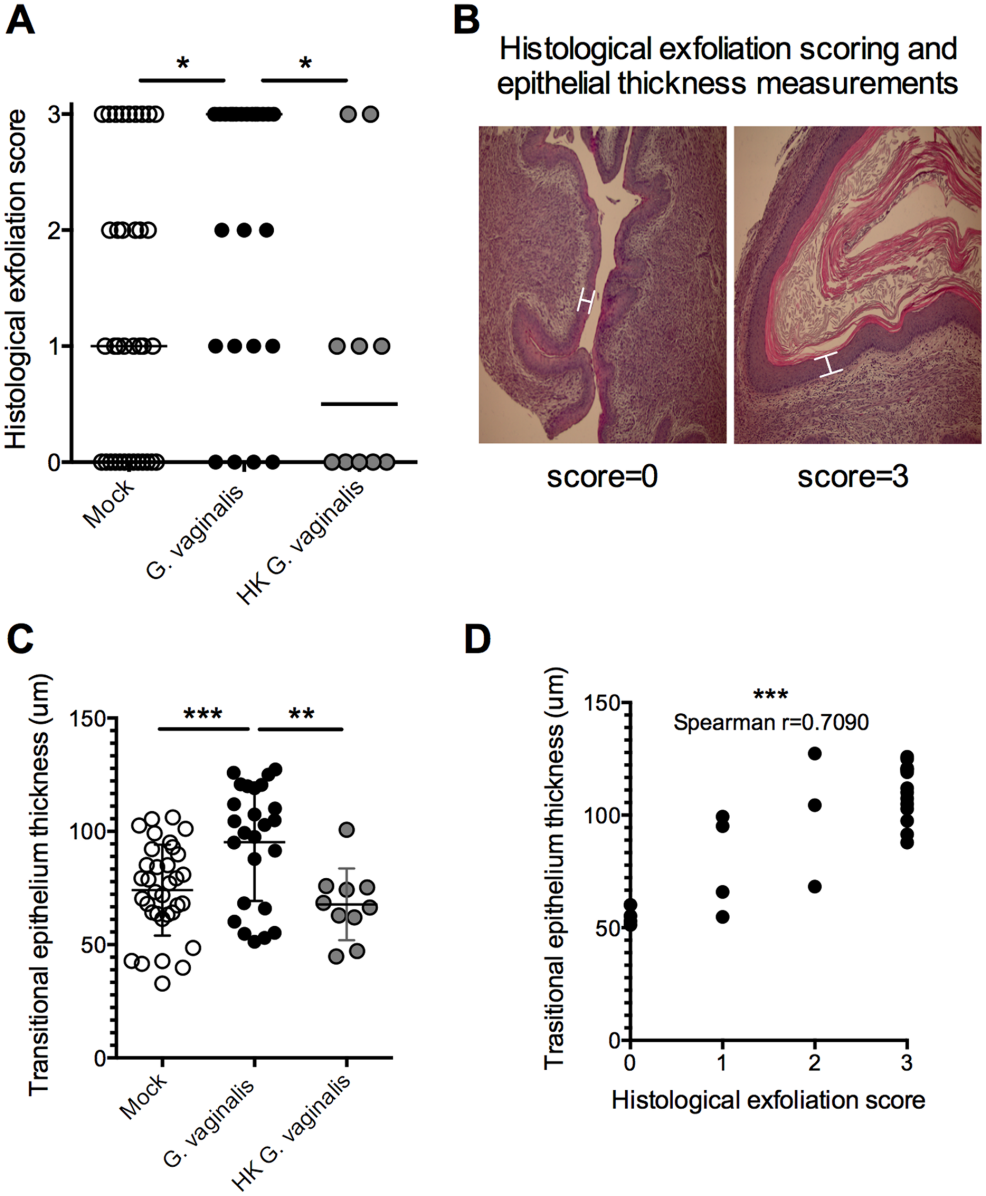
*G. vaginalis* induces a robust histological epithelial exfoliation and proliferation response. (A) Histological exfoliation scoring. H&E stained, formalin-fixed, paraffin-embedded vaginal tissue sections from the 24 hpi time point were assessed for evidence of epithelial exfoliation (eosin-rich layers of epithelial cells superficial to the transitional epithelium) and assigned a numerical value from 0–3, with 0 = none and 3 = very robust. The Kruskal-Wallis test was used to for statistical evaluation of differences between groups (P = 0.0161). For pairwise comparisons, post-hoc testing was performed using the Mann-Whitney U-test, with significance indicated in the figure. *P<0.05. Upon conservative correction for multiple comparisons with Bonferroni-Holm, the non-parametric tests remain significant. (B) Representative images of epithelial scoring, with white, capped lines representing the measured thickness of the transitional epithelial layer shown in (C). (C) Using the same samples from (A), average thickness of the transitional epithelium was determined from five measurements per sample using StreamStart® software. Results in each graph are meta-data from 4 independent experiments (mock n = 36, *G. vaginalis* n = 25, heat-killed (HK) *G. vaginalis* n = 10). The data passed the D’Agostino & Perason omnibus normality test. Therefore, a one-way ANOVA was used detected significant differences between groups (P = 0.0004), followed by post-hoc pairwise comparisons using the unpaired t-test. **P<0.01; ***P<0.001. Again, differences remained significant with the conservative Bonferroni correction. (D) Transitional epithelium thickness values from (C) were plotted against exfoliation scores from (A) and Spearman correlation was determined. ***P<0.001.

While *G. vaginalis* did not elicit a robust inflammatory response, we observed that the epithelial surfaces of *G. vaginalis* infected mice displayed evidence of epithelial cell exfoliation (see **Fig. 1C**). To gain a semi-quantitative perspective of this phenotype, we scored the degree of histological exfoliation in slides that were blinded to the observer, with 0 being none and 3 being very robust (see **Fig. 5B** for representative images). Vaginal sections from *G. vaginalis*-infected mice had significantly higher exfoliation scores compared to mock-infected controls (**Fig. 5A**). Additionally, *G. vaginalis* infection resulted in increased thickness of the transitional epithelium (**Fig. 5C**), which correlated positively with exfoliation score (**Fig. 5D**), suggesting that there may be increased epithelial proliferation in response to the surface exfoliation. Inoculation of heat-killed *G. vaginalis* did not result in a significant increase in either epithelial exfoliation score or thickness (**Fig. 5A&C**), demonstrating that live bacteria are required to induce this response.

**Figure 6:**
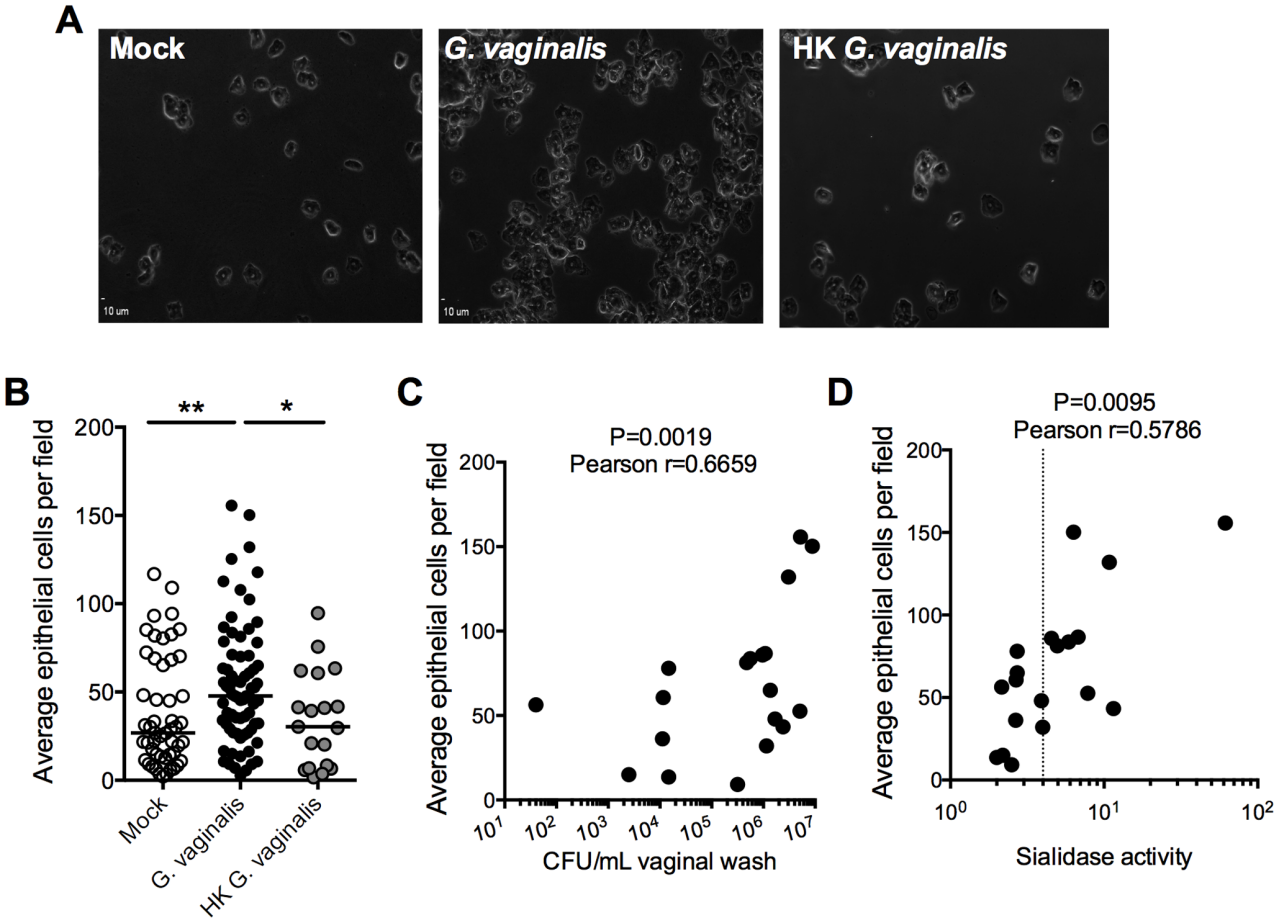
*G. vaginalis*-induced exfoliation is evident in vaginal washes and correlates with vaginal titers and sialidase activity. (A) Representative images of phase contrast light microscopy fields of vaginal wash wet mounts from mock, *G. vaginalis* and HK *G. vaginalis* infection groups, as indicated, used in (B) to enumerate epithelial cells. (B) The average numbers of epithelial cells per field of view were calculated from 5 images per sample. Results are meta-data from 4 independent experiments (mock n = 51, *G. vaginalis* n = 74, HK *G. vaginalis* n = 19). The Kruskal-Wallis test was used to for statistical evaluation of differences between groups (P = 0.0065). The Mann-Whitney U-test was used for post-hoc pairwise comparisons, with significance indicated in the figure. **P<0.01; *P<0.05. Differences remained significant with the conservative Bonferroni-Holm correction for multiple comparisons. (C) and (D) Epithelial counts were plotted against their corresponding vaginal wash CFU (C) or sialidase activities (D) and Pearson correlations were determined.

As an additional assessment of this apparent exfoliation response, we examined vaginal washes by wet mount light microscopy. Consistent with our observations of H&E stained vaginal sections, vaginal washes from both mock and *G. vaginalis* infected mice contained predominantly epithelial cells with very limited, if any, leukocytes (representative images shown in **Fig. 6A**). We enumerated epithelial cells, again in a blinded manner, and found that *G. vaginalis* infected mice had significantly higher numbers of epithelial cells in vaginal washes compared to mock-infected animals or those exposed to heat-killed *G. vaginalis* (**Fig. 6B**). Finally, the degree of epithelial exfoliation in mice infected with *G. vaginalis* correlated positively with both vaginal wash CFU and sialidase activity (**Fig. 6C–D**), consistent with this response being relative to infectious burden. Together these results show that *G. vaginalis* JCP8151B induces a robust vaginal epithelial exfoliation response in a murine vaginal infection model.

### Epithelial Exfoliation as a Clinical Feature of Bacterial Vaginosis

**Figure 7:**
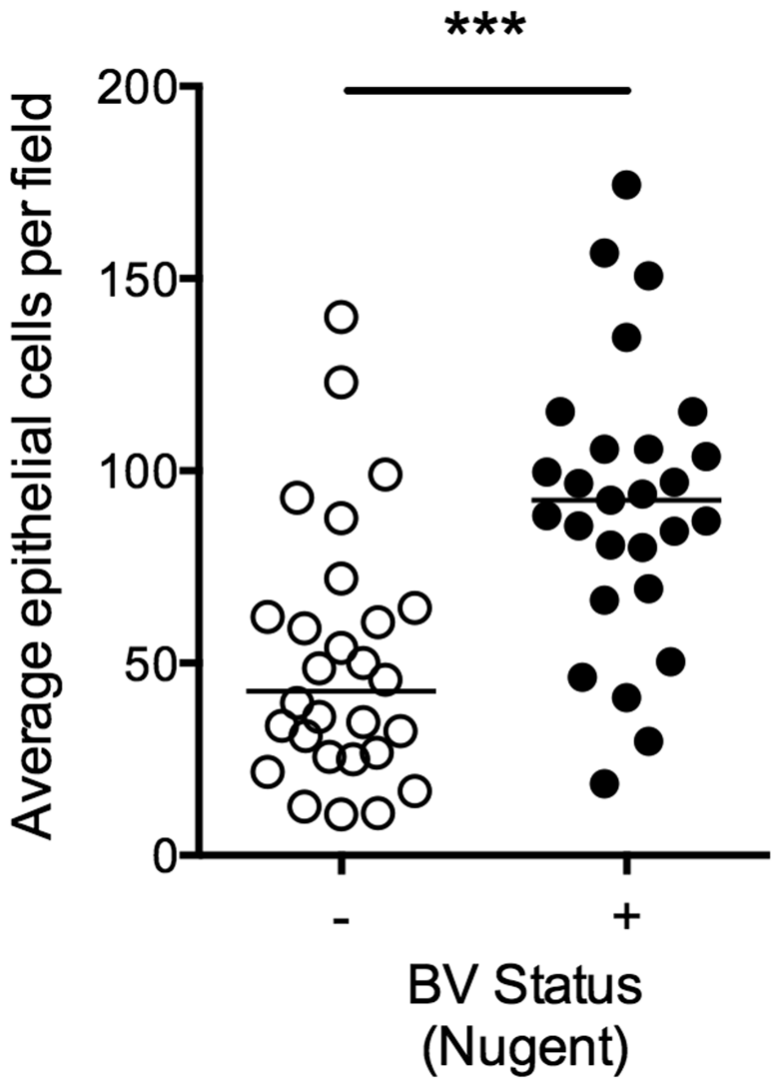
Human clinical samples display evidence of epithelial exfoliation associated with bacterial vaginosis. Heat-fixed, Gram stained slides were prepared from fresh human vaginal swabs and classified as BV (+) or no BV (−) by Nugent scoring (scores 7–10 and 0–3 respectively). The average number of epithelial cells per field of view was calculated from 3 images per sample. BV (−) n = 28, BV (+) n = 27. The Mann-Whitney U-test was used for statistical evaluation of differences between groups. ***P<0.0001.

Pathogen induction and blockade of host epithelial exfoliation responses has been described in other murine urogenital infection models. For example, uropathogenic *E. coli* (UPEC) induces exfoliation of superficial umbrella cells lining the bladder, which is thought to be consistent with the progression of acute UTI in humans [59]–[62]. However, *N. gonorrhea* has been shown to block vaginal epithelial exfoliation through interactions with human specific receptors (carcinoembryonic antigen-related cell adhesion molecules, CEACAMs) [63], [64]. Although the presence of epithelial clue cells is a well-established clinical feature of BV, we found *no* examples in the literature measuring whether the relative number of epithelial cells in clinical specimens is increased in women with BV compared to those with normal flora. To determine whether the increased number of epithelial cells seen in our murine model is a verifiable feature of human BV, we performed microscopic enumeration of vaginal epithelial cells on slides prepared from human vaginal swabs from women with (+) and without (−) BV as defined by Nugent score (7–10 and 0–3 respectively) (**Fig. 7** **)**. Consistent with the murine model, significantly higher levels of epithelial cells were observed on slides prepared from specimens of women with BV compared to women with normal flora (**Fig. 7**). These results strongly suggest that an epithelial exfoliation host response occurs in the clinical setting of BV.

## Discussion

Bacterial vaginosis (BV) is a common vaginal condition in women [1], [13] and is associated with increased risk of sexually transmitted infection and adverse pregnancy outcomes, including preterm birth [13]–[22]. Despite its prevalence, the etiolog(ies) of BV symptoms and complications are poorly understood. There is an obvious and dramatic shift in the overall vaginal flora from a *Lactobacillus*-dominant state to one overrun by high titers of Gram-negative anaerobes and *Actinobacteria*. A handful of bacteria have come to be known as BV-associated bacteria, including *G. vaginalis.* However the contribution of these BV-associated bacteria to the overall disease state is largely undefined. A significant contributing factor to this dearth of understanding is the absence of relevant small animal models.

We found a few recent reports in the literature describing murine vaginal inoculation with *G. vaginalis.* Two of these papers investigated the effects of *Lactobacillus* probiotic strains on *G. vaginalis* colonization in an outbred mouse strain [54], [55]. However, the methods of isolation and enumeration of *G. vaginalis* vaginal titers in these papers were only loosely described. For example, *G. vaginalis* recovered from infected mice were reported as a percentage of the no probiotic control group rather than an absolute enumeration of recovered colony forming units, hampering assessment of the overall bacterial load. Although possibly explained by a difference in mouse strain used or conditions of the housing or breeding facility, there was also no reference to the incidence of contaminating vaginal flora, which we found invariably present in our mouse model. In addition to these two studies, there is one report of vaginal *G. vaginalis* infection in gnotobiotic mice [65], which naturally circumvents the issue of contaminating flora.

Here we describe a murine vaginal infection model with *G. vaginalis* that is, to our knowledge, the first to recapitulate key BV phenotypes. Technically speaking, this model is rather straightforward and very similar to infection models utilized for other vaginal pathogens. However, the model described here is distinguishable by the fact that previous studies of *G. vaginalis* infection in mice did not investigate BV-related phenotypes *in vivo*. Furthermore, we took multiple measures to ensure accuracy of our *G. vaginalis* titer enumeration, including 1) generating a streptomycin-resistant (Sm^R^) isolate, and 2) confirming by PCR that bacteria re-isolated from mouse vaginal washes were *G. vaginalis*. Vaginal inoculation with *G. vaginalis* was sufficient to yield **1)** vaginal sialidase activity; **2)** epithelial cells with adherent bacteria (reminiscent of clue cells); **3)** epithelial exfoliation in the absence of an inflammatory infiltrate. Together, results from this model demonstrate that *G. vaginalis* likely plays a role in generating these clinical features observed in humans and each is discussed in further detail below.

### Sialidase Activity

Sialidase activity is rarely detected in women with normal flora [19], [66], [67]. In pregnant women, sialidase activity has also been independently correlated with increased risk of chorioamnionitis and PTB [19], [68]. Production of sialidase by isolated BV-associated bacteria grown in culture strongly suggests that BV-associated sialidases are bacterial in origin [69], [70]. Bacterial sialidases have been characterized as virulence factors in bacterial infections of various mucosal sites [71]. Our previous *in vitro* studies have highlighted the potential role of *G. vaginalis* sialidase in the deglycosylation and degradation of host glycoproteins [72]. In our murine model, the level of sialidase activity correlates with vaginal *G. vaginalis* titers and sialidase positive mice are more likely to develop uterine horn infection. These data suggest that higher vaginal titers are more likely to result in ascending infection. Although further studies are required, these results may also suggest potential role for sialidase in the mechanisms facilitating ascending infection. Consistent with this idea, sialidase activity levels in pregnant women correlated directly with increased risk of chorioamnionitis and preterm birth [7], [68].

### *G. vaginalis* and the Formation of Clue Cells

It has long been established that BV bacteria interact with epithelial cells. In fact, the presence of exfoliated clue cells is a qualitative diagnostic feature of the disease [73], [74]. It has previously been suggested that *G. vaginalis* may be responsible for clue cell formation, since it was detected on the surface of exfoliated vaginal epithelial cells more frequently and at higher levels than the BV-associated anaerobes *Mobiluncus*, *Bacteroides*, and *Fusobacterium* [39]. A more recent high-resolution phylogenetic study examining correlations between different species of BV bacteria and clinical features revealed that *G. vaginalis* is positively associated with the presence of clue cells [75]. *G. vaginalis* has also been shown to interact with a vaginal epithelial cell line [36] and epithelial cells present in human clinical samples [40], [76]. We demonstrated, using fluorescently labeled bacteria, that *G. vaginalis* interacts with murine vaginal epithelial cells, forming clue-like cells *in vitro*. Labeled *G. vaginalis* was also detected on the surface of epithelial cells recovered from vaginal washes of mice following infection with *G. vaginalis*. Together these data provide evidence that our murine model of *G. vaginalis* infection displays this important feature of BV and provides an avenue for investigating clue cell formation *in vivo*.

### *G. vaginalis* Induces Epithelial Exfoliation in the Absence of Overt Inflammation

BV is characterized by a heavy overgrowth of *Actinobacteria* and Gram-negative anaerobes but a surprising absence of the type of inflammatory infiltrate seen in other urogenital infections such as gonococcal infection [77], [78] or urinary tract infection [46], [79], [80]. Epithelial shedding, or exfoliation, appears to be a common mechanism of protection employed by mucosal surfaces [81], [82]. *G. vaginalis* vaginal infection in mice produced a robust exfoliation response that correlated directly with vaginal titers and vaginal sialidase activity.

Consistent with the lack of overt inflammation in women with BV [83], our histological and wet mount microscopy analyses of vaginal specimens from mice infected with *G. vaginalis* displayed no signs of polymorphonuclear leukocyte (PMN) recruitment. Even though estradiol treatment suppresses the influx of PMNs that naturally occurs in mice after ovulation [84], literature evidence has shown that PMN recruitment to the vagina can still occur upon infectious challenge in C57/Bl6 mice [85]. Although no quantitative data was given, Teixeira et al. reported the presence of ‘inflammatory lesions’ in the vaginas of gnotobiotic mice infected with *G. vaginalis* [65]. It is reasonable to suspect that indigenous microflora in the vagina may contribute to host innate immune responses, as has been shown in the gut [86], [87]. This may influence inflammatory responses to *G. vaginalis* in mice lacking endogenous flora. Further studies are required to provide a better understanding of the role of *G. vaginalis* in the apparent suppression of inflammatory responses that occurs during BV. Recent biochemical and genomic investigations have revealed that *G. vaginalis* isolates can be remarkably diverse [88]–[91], perhaps allowing different host responses to various strains of *G. vaginalis*.

### Epithelial Exfoliation as a Measurable Clinical Feature: Implications for Understanding BV

Epithelial exfoliation has long been discussed in the BV literature, most often with regard to clue cells. Although there are countless references to “increased epithelial exfoliation” in BV, we found no examples of quantitative analysis in a defined experimental or clinical setting. We present evidence that an increased vaginal epithelial exfoliation response, a robust feature of the *G. vaginalis* animal model described here, is also apparent in Gram-stained images of vaginal fluids from women with BV (Nugent 7–10) compared to normal controls (Nugent 0–3). Our analysis of clinical samples provides the first concrete evidence to classify an exfoliation response as a BV phenotype.

The modest but significant increase in the number of exfoliated epithelial cells in women with BV may be a beneficial response if it removes adherent potential pathogens and when surface epithelial layers can be replenished. However, excessive exfoliation may promote access to underlying tissue, which may facilitate the establishment of BV-associated bacteria and increase the risk of secondary infection. In fact, BV is known to be associated with increased risk of certain sexually-transmitted infections (STI) [18], [92], [93] and some vaginal pathogens can take advantage of the exfoliation process to facilitate access to underlying tissue. For example, *T. vaginalis* causes contact-dependent cytotoxicity upon adherence to vaginal epithelial cells, thereby leading to exfoliation and erosion of the epithelium. It has been suggested that this may allow trichomonads into extracellular matrix and basement membrane sites within the vaginal tissue [45]. It is possible that removal of the outer epithelial cell layer by *G. vaginalis* provides a niche for formation of an adherent biofilm, which has been observed in vaginal biopsies from women diagnosed with BV [42]. Interestingly, BV correlates with *T. vaginalis* infection, therefore it is conceivable that exfoliation induced by *G. vaginalis* and *T. vaginalis* could be mutually beneficial and may also impact other vaginal organisms. Interestingly, some vaginal microbicides have been shown to have paradoxical effects, actually increasing susceptibility to HIV and other sexually-transmitted pathogens. This increased susceptibility was found to be coincident with rapid exfoliation and re-growth of epithelial cell layers [94]. These previous findings are consistent with the idea that epithelial exfoliation in BV may contribute to increased STI risk. Future studies should examine whether epithelial exfoliation may contribute to the overall risk of secondary infections associated with BV.

We found a single reference in the literature that performed quantitative analysis of vaginal epithelial shedding (by counting epithelial cells present in vaginal lavage samples). Interestingly, this study uncovered a link between vaginal epithelial exfoliation and smoking [95], a behavior that has been shown to be associated with BV [96]. If exfoliation promotes vaginal colonization by BV bacteria, it is tempting to speculate that the link between smoking and BV could be explained, at least in part, by the initiation of epithelial exfoliation that occurs in smokers. Ultimately, the downstream ramifications of epithelial exfoliation for the overall pathophysiology of BV remain to be explored and the new murine model presented here provides a valuable tool for these investigations.

### *G. vaginalis* as a Pathogen

Since it was first described, there has been vigorous debate in the literature regarding the role of *G. vaginalis* in BV. *G. vaginalis* is one of the most frequently isolated bacterial species from women with BV. Consistent with the notion of *G. vaginalis* as a potential pathogen, strains identified as *G. vaginalis* have been isolated from placenta, amniotic fluid, and blood [32]–[34]. *G. vaginalis* has also been implicated in uterine infections and development of endometritis [97]. Results from a comparison of epithelial adhesion, cytotoxicity and biofilm formation between several BV associated bacteria suggested that *G. vaginalis* may be more virulent than other species associated with the disease [37]. The observation that *G. vaginalis* produces BV phenotypes in our murine model, in the absence of other BV-associated bacteria, emphasizes its likely role in BV etiology. However, the main controversy appears to lie in the fact that *G. vaginalis* can also be detected from women with normal flora [2], [27], [29]–[31]. We argue that the presence of *G. vaginalis* in healthy individuals does not constitute a basis for disregarding this bacterium in the causes and complications of BV. Just as “healthy” people can be asymptomatic carriers of such pathogens as *Streptococcus pneumoniae* [98], Group A *Streptococcus* [99], [100], *Haemophilus influenzae* [98] or *Clostridium difficile* [101], carrier states may also exist for *G. vaginalis.* Indeed, recent genomic and phenotypic studies support the hypothesis that variations in bacterial strain virulence, titers, and/or windows of host susceptibility may bring a colonization state to a state of pathogenesis [38], [88], [91], [102], [103].

In summary, our results demonstrate for the first time that *G. vaginalis* is sufficient to yield key BV phenotypes in an animal model. The quantitative experimental methods described here show that infection with *G. vaginalis* leads to vaginal sialidase activity, bacterial adherence to vaginal epithelial cells, and a robust epithelial exfoliation response that we demonstrate as a relevant clinical feature of BV in quantitative, controlled experiments. These data provide strong evidence that *G. vaginalis* can play an active role in generating clinical features associated with BV. The phenotypic parallels to human BV displayed in the murine system provide a new experimental tool with great potential to expand our understanding of *G. vaginalis*-host interactions in the vagina.

## Materials and Methods

### Ethics Statement

Vaginal swabs were collected as part of the Contraceptive CHOICE project [104] according to protocols approved by the Washington University Institutional Review Board (IRB ID# 201108155). Mouse experiments were carried out in strict accordance with the recommendations in the Guide for the Care and Use of Laboratory Animals. The protocol was approved by the Animal Studies Committee of Washington University School of Medicine (Protocol Number: 20110149).

### Bacterial Strains and Growth Conditions

*Gardnerella vaginalis* clinical isolate JCP8151B (GenBank JX860320) was obtained from a vaginal swab from a woman with BV (based on Nugent score) obtained in accordance with IRB-approved protocols in collaboration with the Washington University Contraceptive CHOICE Project (IRB ID# 201108155). The vaginal swab was transported from the clinic to the lab using Port-A-Cul™ pre-reduced anaerobic transport media tubes (BD). Tubes were brought into a Vinyl anaerobic airlock chamber (Coy Products) under an atmosphere maintained at approximately 1% hydrogen and 0 ppm oxygen. Within 24 hours and the swab was used to inoculate “*Gardnerella* semi-selective media,” (agar plates with 5% defibrinated sheep blood, 10 mg/L colistin, 10 mg/L nalidixic acid, and 4 mg/L amphotericin B). Plates were pre-incubated in the chamber for at least 16 hours for equilibration to anaerobic conditions and were incubated anaerobically post-inoculation at 37°C for 24–48 hours. Translucent pinpoint colonies were isolated, and candidate *G. vaginalis* strains were tested by diagnostic PCR using primers previously reported to be specific for *G. vaginalis* (forward primer GGGCGGGCTAGAGTGCA and reverse primer GAACCCGTGGAATGGGCC) [56]. Additional validation of the *G. vaginalis* identity was obtained by sequencing 16S rDNA. Full details of this strain are being reported in a separate manuscript.

A spontaneous streptomycin resistant mutant, JCP8151B-Sm^R^ #4, was isolated by plating JCP8151B (concentrated from a 2 day, 25 mL NYC-III culture) on *Gardnerella* semi-selective media +1 mg/mL streptomycin and selecting resistant colonies after incubating anaerobically at 37°C for 72 hours. We confirmed the streptomycin resistance in this isolate was due to point mutation of the Rpsl gene (as that reported for other bacteria) by amplifying *G. vaginalis* Rpsl with primers rpsL F2 (CATGGTTTAAGGTGTGCTG) and rpsL R (GTTAATCAACTGAGCCACG) and sequencing using rpsL F2. To confirm that the point mutation did not result in any apparent growth defects or changes in sialidase activity, JCP8151B and JCP8151B-Sm^R^ #4 were grown anaerobically overnight in 5 ml NYC-III medium at 37°C, then diluted to OD_600_ of 0.1 in 5 mL NYC-III medium. A 25 µL aliquot of each bacterial suspension was analyzed for sialidase activity as described below. The remaining bacterial suspension was incubated anaerobically at 37°C for 24 hr to monitor growth. Aliquots were removed at 0, 1, 3, 6 and 24 h, serial diluted and plated on *Gardnerella* semi-selective media to enumerate colonies. Results for sialidase activity and growth curves were indistinguishable between JCP8151B and JCP8151B-Sm^R^. The JCP8151B-Sm^R^ isolate was used for murine infection model experiments described below. Control experiments (as dictated in figures and figure legends) treated a parallel group of mice infected with JCP8151B-Sm^R^ that was heat-killed by incubation of the bacterial inoculum at 80°C for 10 min.

### Murine Vaginal Infection Model

Female C57/Bl6 mice (6–8 weeks) were injected intraperitoneally with 0.5 mg β-estradiol in 100 µL filter-sterilized sesame oil three days prior to and on the day of inoculation. Mice were anaesthetized with isofluorane and inoculated vaginally with ∼5×10^7^ CFU *G. vaginalis* in 20 µL sterile PBS (OD_600_ = 5.0). Vaginal washes were collected by flushing vaginas with 50 µL sterile PBS using a P200 pipet (GeneMate), pipetting up and down 10x, followed by rinsing into an additional 10 µL PBS in a sterile 1.5 mL Eppendorf tube. *G. vaginalis* titers were determined from washes by preparing 10-fold serial dilutions in PBS (in the anaerobic chamber) and spotting 5 µL of each dilution in quadruplicate onto 1 mg/mL streptomycin selection plates (either Gardnerella semi-selective media or NYC-III agar). Colonies were then enumerated and reported as recovered colony forming units (CFU) per mL of vaginal fluid. Vaginal washes were also analyzed for sialidase activity and epithelial exfoliation as described below.

Mice were sacrificed at 24 hpi or 72 hpi to harvest vaginas and uterine horns. One uterine horn and half of the vagina (bisected longitudinally) from each mouse was homogenized followed by serial dilution and plating as for vaginal washes. Colonies were enumerated and reported as CFU per gram of tissue. The remaining vaginal tissue and uterine horn from each mouse were fixed in 10% buffered formalin phosphate at room temperature followed by paraffin embedding. Histological slide preparation and H&E staining were performed by the Washington University School of Medicine Histology Core.

### Sialidase Activity Assays

Vaginal wash samples collected as described above (25 µL) were diluted 1∶2 with 100 mM sodium acetate pH 5.5 containing 300 µM 4-methylumbelliferyl-Neu5Ac (50 µL). Substrate hydrolysis was monitored using a Tecan M200 plate reader.

### *G. vaginalis* Interaction with Murine Vaginal Epithelial Cells in vitro and in vivo

*G. vaginalis* JCP8151B was fluorescently labeled with Rhodamine B isothiocyanate (RBITC; Aldrich 283924). RBITC was prepared fresh at 0.2 mg/mL in 20 mM HCl and 5 µL of this stock was added to 500 µL *G. vaginalis* JCP8151B in sterile PBS (prepared as described for mouse experiments above). The bacteria were then incubated anaerobically at 37°C for 30 min, centrifuged, resuspended in 500 µL NYC-III and allowed to recover with an additional 30 min, 37°C anaerobic incubation. Finally, the labeled bacteria were washed twice with PBS and resuspended in 500 µL PBS for inoculation of epithelial cells, as described below. For each experiment a “mock” labeled sample, lacking bacteria, was prepared in parallel.

While *G. vaginalis* was being labeled, vaginal washes were collected from β-estradiol-treated mice, as described above. Washes were pooled and spun at 300 g for 5 min to collect epithelial cells. Epithelial cells were then washed 3 times with sterile PBS to remove the majority of endogenous flora and then distributed in 50 µL aliquots into 1.5 mL eppendorf tubes. RBITC-labeled *G. vaginalis* or the “mock” labeled sample (5 µL) were added to the epithelial cells and the samples were rotated at 37°C for 3 hours. Finally, epithelial cells were washed twice to remove unassociated bacteria and then visualized on an Olympus BX61 confocal fluorescent microscope using SlideBook 5.0 software.

For *in vivo* analyses, *G. vaginalis* was labeled with RBITC as described for *in vitro* experiments and then inoculated vaginally into β-estradiol-treated mice as described above. An additional group of mice was inoculated with a “mock”-label preparation containing no bacteria. At 4 hpi, mouse vaginas were washed with 50 µL PBS and epithelial cells were visualized by confocal fluorescent microscopy as described above.

### Analysis of Murine Epithelial Cell Exfoliation

H&E stained mouse vaginal histology sections collected above were visualized on an Olympus BX61 microscope to assess the degree of inflammation (24 hpi and 72 hpi time points) and epithelial exfoliation (24 hpi time point). Images were captured and epithelial thickness was measured using StreamStart® software, with averages calculated from 5 measurements per vaginal section.

For assessment of epithelial exfoliation in mouse vaginal washes, wet mounts were prepared with 5 µL vaginal wash (from the 24 hpi time point) and visualized by phase contrast microscopy using an Olympus BX61 microscope. Five representative images were captured from each specimen (1 per mouse) and epithelial cells were counted from each image to determine an average.

### Clinical Specimen Handling and Analysis of Epithelial Exfoliation

Vaginal swabs (Starplex) were collected as part of the Contraceptive CHOICE project [104)] according to protocols approved by the Washington University Institutional Review Board (IRB ID# 201108155) and underwent Nugent scoring using published methods as previously described [72], [105]. Gram stained slides (the same used for Nugent scoring) were analyzed for epithelial exfoliation by enumerating epithelial cells in three representative images as described for murine samples above.

### Statistical Analysis

GraphPad Prism 5.0 software was used for all statistical analyses presented. The statistical tests used to analyze each set of data are indicated in the figure legends.
